# Why is tobacco control progress in Indonesia stalled? - a qualitative analysis of interviews with tobacco control experts

**DOI:** 10.1186/s12889-020-08640-6

**Published:** 2020-04-19

**Authors:** Putu Ayu Swandewi Astuti, Mary Assunta, Becky Freeman

**Affiliations:** 1 Department of Public Health and Preventive Medicine, Faculty of Medicine, Udayana University, Denpasar, Bali Indonesia; 2 School of Public Health, The University of Sydney, Sydney, NSW Australia; 3 South East Asia Tobacco Control Alliance (SEATCA), Bangkok, Thailand; 4 Prevention Research Collaboration (PRC), Charles Perkins Center, The University of Sydney, Sydney, NSW Australia

**Keywords:** Smoking, Tobacco control, Tobacco privilege, Advocacy, WHO-FCTC, Indonesia

## Abstract

**Background:**

Indonesia shoulders a significant tobacco burden, with almost two million cases of tobacco-related illnesses and more than two hundred thousand tobacco-related deaths annually. Indonesian tobacco control is progressing but lags behind other countries. Our study evaluates factors that contribute to the slow progress of tobacco policy change in Indonesia from the perspective of tobacco control experts (TCEs).

**Method:**

We conducted qualitative interviews with four international and ten national TCEs, who have been active in tobacco control for at least 5 years. Our interview guideline included questions on the current tobacco control situation in Indonesia and explored reasons why tobacco control is progressing so slowly. The interviews were conducted either in English or Bahasa Indonesia, recorded and then transcribed verbatim. We conducted a thematic analysis based on five core causal factors for policy adoption: institutions, networks, socio-economic factors, agendas and ideas.

**Results:**

The multistage delay of tobacco policy adoption is principally due to political structures and policy hierarchy, complex bureaucracy, unclear roles and responsibilities, and a high degree of corruption. The low bargaining position and lack of respect for the Ministry of Health also contributes. There are contrasting frames of tobacco as a strategic economic asset and tobacco control as a sovereignty threat. There is an imbalance of power and influence between well entrenched and resourced tobacco industry networks compared to relatively young and less established tobacco control networks. The policy agenda is likely influenced by the privileged position of tobacco in Indonesia as a socially acceptable product with high consumption. There are constraints on transferring ideas and evidence to successful policy adoption.

**Conclusion:**

Tobacco companies have substantially influenced both policy decisions and public perceptions, signifying a power imbalance within the government system and broader networks. Acceding to and enforcing the World Health Organization- Framework Convention on Tobacco Control (WHO-FCTC) would enable the Indonesian government to shift the power imbalance towards public health stakeholders. Tobacco control advocates must enhance their network cohesion and embrace other community groups to improve engagement and communication with policymakers.

## Background

The adoption of the World Health Organization Framework Convention on Tobacco Control (WHO-FCTC) marked the global consensus of the urgency to control the alarming tobacco epidemic. Ratifying countries recognised the detrimental consequences of tobacco use on health, socioeconomic status, and the environment [1]. Indonesia is the only country in the Asia Pacific region that has yet to ratify the treaty despite the significant tobacco burden within the country.

Indonesia is home to almost one hundred million smokers, where 33.6% of the adult population and 19.4% of young people age 13–15 years are smokers [2, 3]. A 2018 national survey showed smoking prevalence among youth 10–18 years old was 9.1%, a significant increase from 2013 at 7.2%, [4] and far higher than the government target of 5.4% in 2019. Indonesia suffers an economic loss of US$ 45.9 billion due to tobacco use, with almost 2 million cases of tobacco-related illnesses and 230,862 tobacco-related deaths in 2015 [5].

Conversely, tobacco industry proponents argue that tobacco control will cause economic harm through loss of revenue and depriving tobacco farmers and industry workers from earning a living [6]. A study of 1350 smallholder tobacco farmers in Indonesia showed that tobacco farming is not profitable for the farmer, most farmers are poor, and many suffer from green tobacco disease [7]. Similar to other settings, smoking prevalence in Indonesia is higher among the lower socio-economic quintiles [8]. Cigarettes are the second highest household expenditure after rice in both urban and rural area of Indonesia [9]. Cigarette smoking also contributes to long term family deprivation, especially children, due to reduced access to proper nutrition and education which in turn makes it harder for the children to escape the poverty cycle as they get older [10].

Tobacco control advocates in Indonesia have been working at both the national and sub-national level with some promising results, with several sub-national governments adopting smoke-free regulations and partial tobacco advertising bans. At the national level, with the noted exception of the adoption of a 40% pictorial on-pack health warning, no significant progress has been made. Indonesia remains one of the few countries that broadcasts cigarette advertisements on television, and its cigarette tax is amongst the lowest in the world. The size of the cigarette business in Indonesia seems to be an essential factor in this slow progress. Indonesia is the second largest cigarette market in the world, with an overall retail volume of 316.1 billion sticks per year in 2016 [11].

While there is evidence of complicities between the tobacco industry and governments in almost all South East Asian (ASEAN) countries, [12] the level of tobacco industry interference in policy making in Indonesia is the highest [13]. Additionally, tobacco companies present themselves as ethical corporations that contribute to government revenue, enact so-called corporate social responsibility programs, and award sponsorships [14]. However, the Indonesian government must increase its commitment to tobacco control measures if it hopes to stem the rising epidemic and to harness the benefits of the predicted demographic dividend, where the working age group will reach 70% of the population by 2030 [15].

Understanding what tobacco control experts (TCEs) perceive the underlying factors stalling and blocking tobacco control policy reforms in Indonesia provides insight for future advocacy efforts. Our study evaluates factors that contribute to stagnant tobacco policy changes in Indonesia.

## Method

### Study design

This study is a qualitative exploration of TCEs’ perspectives of factors that influence the slow progress of tobacco control policy in Indonesia.

### Respondents

Our respondents were TCEs defined as individuals involved in tobacco control advocacy, research or a tobacco control focused organization, for at least 5 years, or who have published works or been quoted in the media regarding tobacco control issues, and do not have any connection to the tobacco industry. We invited in total 16 (5 international and 11 national TCEs), and finally interviewed four international and ten national TCEs, who have been working in tobacco control ranging from 5 to 40 years.

The selection of the international interviewees was based on their knowledge of Indonesian tobacco control. The national tobacco control experts represented academics, community organisations, and national and sub-national government stakeholders. Their expertise included: law, media, health economics, public health, human rights, and youth advocacy. We sent the interview invitation by email, described the purpose of the study and provided the participant information statement. Once they agreed to participate, an interview time was arranged and conducted in September/October 2018.

### Data collection

Interviews were conducted through face-to-face meetings and video calls, each lasting up to 60 min. We used an interview guide that contained a set of initial questions (Supplementary file 1), which were then further probed based on interviewee responses. The guide included questions on the TCEs perspectives of the current tobacco control situation in Indonesia and reasons why tobacco control is not progressing well. The interviews were conducted either in English or Bahasa Indonesia, recorded and then transcribed verbatim. We ceased conducting interviews when we were satisfied that we had reached data saturation and had collected a range of views.

### Theoretical framework

In order to explore factors that influence the slow progress of tobacco control, we adopted the five core causal factors of policy processes proposed by Peter John [16], and adapted by Cairney et.al for tobacco control [17]. These five factors, defined in Table 1, include institutions, networks, socio-economic factors, agendas and ideas [17]. We selected these factors to systematically understand the data, and to discuss relationships between these factors in the context of Indonesian tobacco control policy adoption and policy changes.

**Table 1:** Definition of five core causal factors^a^

Causal Factors	Definition
Institutions	The authority, rules, organisational values and the way the organisation act and decide tobacco control policy
Agendas	Framing of the problem and importance of tobacco as policy issue.
Networks	Pressure participants and shift of power among them.
Socioeconomic factors	The role of socioeconomic factors and its contribution to the power shift and agenda changes
Ideas	The role of scientific knowledge and the influence of transfer of idea to policy

^a^Adapted from Cairney et.al [17]

### Analysis

We conducted a step-wise thematic analysis [18]. First, we conducted data immersion, where we read and re-read the data, to become immersed and familiar with its content; this was followed by a line by line coding of all interview content, conducted by author PASA. We created codes that indicated important features of the data that might be relevant to our study aim of examining policy factors. Our third step was to generate, review and refine themes, we examined our codes to identify patterns of meaning as potential themes. We used the five causal factors described in Table 1 as our initial guide to identifying themes, and then we checked the themes against the dataset, and developed more detailed sub-themes. We refined the themes and sub-themes to build a coherent story based on our study aims. The second author (BF) reviewed the final set of themes and sub-themes to check that it was aligned with the data. We defined the scope and focus of each sub-theme and assigned each an informative name (Table 2). The final step was writing up the analysis, we weaved the analytic narrative, the themes and data extracts, and contextualised the analysis in relation to existing literature and the Indonesian context. We supported the results with direct quotes from the interviews, some of which are translated from Bahasa Indonesia. We have also provided the respondent number and position of the quoted TCEs.

**Table 2:** Final Themes and sub-themes

Themes and Sub-themes	Definition
I. Institutions	Actors that are responsible for establishing tobacco control policy
I.1. Political structure and type of policy: multistage delay	Barriers to policy adoption and implementation due to government and parliament structures and relationships.
I.2. Government bureaucracy and corruption	Loopholes within government that are prone to tobacco industry interference, including the complexity of government roles and responsibilities and degree of corruption.
I.3. Role of Ministry of Health (MoH): low bargaining position	Perceived role and position of MoH in efforts to strengthen tobacco control
II. Agenda	Framing of tobacco and tobacco control in Indonesia
II.1 Political economy framing of tobacco: We need the money- we need the employment	Perception of tobacco as an economic commodity and a source of income and employment rather than a threat to health and community prosperity.
II.2. Sovereignty framing: tobacco control is an “outsider” project	Viewing the tobacco control movement as a foreign agenda and a threat to national sovereignty.
III. Networks	Pressure participants and power shifts.
III.1 Tobacco industry network: Established and well-funded	Well established tobacco industry network that infiltrates the policy system with funding and frontline groups
III.2. Tobacco Control Network: Resources and cohesion	Tobacco control is an ongoing movement with potential competition and limited resources.
IV. Socio-economic factors	The exclusive positioning of tobacco in the community contributes to its exception within the law and barriers to policy change
IV.1. Social acceptability of tobacco: cigarettes are legal, and smoking is normal	Community views toward cigarettes, smoking, and tobacco companies are influenced by high smoking rates and tobacco industry tactics
IV.2 Tobacco exception in the law	Privilege and exception given to tobacco within the law compared to alcohol, which potentially hampers the advancing of tobacco control
V. Ideas and transfer of ideas	Views toward the availability of evidence and best practices, and perceptions of the barriers to transfer of evidence to policy makers.

## Results

### Institutions

This theme describes institutional barriers to establishing tobacco policies in Indonesia. National policy changes may be influenced by domestic and inter-governmental institutions. For this analysis, we focused on documenting domestic institutions that may impact on tobacco control policy. We identified three sub-themes including: 1) political structure and type of policy**:** multistage delay, 2) bureaucracy and degree of corruption, and 3) role of Ministry of Health (MoH).

#### Political structure and type of policy: multistage delay

The Indonesian government system is a presidential democracy, where the president serves the executive role as both head of state and government. Administratively, Indonesia is comprised of provinces, and districts/cities [19]. These sub-national governments gained considerable autonomy over their jurisdictions after the adoption of the decentralisation policy in 1999 [20].

The TCEs highlighted weaknesses in the current national-level tobacco control regulation, PP 109/2019 [PP] [21]. The PP is a joint regulation between three ministries and is considered a compromised outcome. Besides the partial nature of most of the tobacco control policies contained within it, the regulation is vague and provides significant loopholes during stages of implementation. Most elements of the PP require further regulatory action at either the national or sub-national level in order to be implemented.

*When the PP was adopted in 2012, many people viewed it as a victory, but when the implementation started, we were not winning at all except for adoption of pictorial health warnings. Smoke-free law, is it automatically adoptable? No! We need a joint decision letter from three ministries so that the sub-national government can create sub-national bylaw. (Respondent 3, Advocate).*

In addition to national level actions, policy making is also taking place at sub-national/provincial and district/city level. Some aspects of the PP were mandated to the sub-national level, [21] which makes sense given the decentralisation of government. The TCEs highlighted this condition as another potential delay, due to the large number, 514 in total, of cities/districts in Indonesia, [19] the lack of political will of some sub-national policy makers, and the potency of tobacco industry interference.

*Unfortunately, that will take time, right? Because if you have 500 cities and then it will take hopefully not 50 years; 50 years means ten cities per year, right? (Respondent 12, International expert)*

*As head of Indonesian Mayor Alliance, I did roadshows to several districts/cities. The result depends on the leader. It is better to start from the area with no tobacco farmers because it is harder if the districts have tobacco farmers. (Respondent 6, Sub-national leader)*.

#### Government bureaucracy and corruption

The TCEs outlined the complexity of bureaucracy and lack of coordination between stakeholders, Ministries, and other government bodies as a significant hurdle to tobacco control progress in Indonesia. Unclear authority and responsibilities of each stakeholder has created delays in adequate implementation of the current regulation. TCEs pointed out that Ministries failed to take responsibility for monitoring cigarette advertisements and would pass to other Ministries/agencies. Coordination between national, provincial and district/city level is not a simple undertaking. District regulations need to be synchronised with provincial governments which furthers complicates the policy making process.

*One of the debates, I remember, who is in charge of cigarette ads, MoH did not consider it was their authority. Who has the authority? It should be under Ministry of Trade, this Minister, that Minister, it has not resolved (Respondent 3, Advocate)*

*Some districts (in East Java) have drafted a smoke free law, which is currently going through a synchronisation stage at the provincial level. At the provincial level, there is a recommendation to postpone the smoke free by-laws, instructed by the governor. The governor stated “smoke free laws should not be discussed for now”. (Respondent 11, Academic)*

The current regulation also mandates that different government agencies handle certain aspects of implementation. However, unclear descriptions within the regulation have allowed agencies to shirk their responsibilities. The TCEs also shared that the PP has crippled the role of the Indonesian Food and Drug Monitoring Agency *(BPOM)*. *BPOM* can issue a warning letter but cannot impose any further sanctions, as other agencies have responsibility for enforcement oversight. For example, the Indonesian Broadcasting Commission *(KPI)* should monitor and enforce any violations of any broadcast media advertisements and sub-national governments are responsible for any outdoor ads.

*So, PP 109 is “gnawing” away the monitoring function of BPOM. The mandate to monitor overall advertising, promotion and sponsorship should be held by BPOM, except outdoor advertisements by the sub-national government. But when the BPOM conducts monitoring, they are only able to write a recommendation letter. (Respondent 4, Advocate).*

This complex bureaucracy, coupled with decentralisation, increases vulnerability to corruption [22, 23]. The TCEs argued that tobacco funds provided may support governments programs or be part of corporate social responsibility (CSR) efforts to halt regulations.

*However, there is another combination, a country with a high degree of corruption. I prefer to say it is political interest rather than personal interest even though it may go into their pocket. It has been stated openly for election, for something like that. It has been publicly said, basically. (Respondent 9, Advocate)*

#### Role of Ministry of Health (MoH): low bargaining position

Significant positive shifts in global tobacco control, especially in developed settings, is partially due to the leadership role of Ministries of Health and the successful framing of tobacco as a pressing public health issue, and not as an economic asset [17]. In low-middle income countries, while Ministries of Health are considered the most active Ministry, most have low influence, with some notable exceptions such as India and Thailand [17].

TCEs highlighted the lack of leadership by the current MoH, the weak position of the MoH as the sole champion of tobacco control, and that it is often undermined by other Ministries. There is only limited cross-government support, notably from the Ministry of Women Empowerment and Child Protection (MWECP).

*And the Ministry of health is the weakest, which should be the most powerful because it is their task and responsibility to reduce (smoking) prevalence. (Respondent 4, Advocate)*

*Even Ministry of Education and Ministry of Youth & sports hesitates to support us, they will say “we need the money for sports and scholarships” (Respondent 14, Government official)*.

Meanwhile, there is active opposition within the government, especially from ministries with direct involvement with tobacco such as the Ministry of Industry, Ministry of Trade and Ministry of Agriculture [6]. The TCE we interviewed who are MoH employees expressed difficulties in negotiating tobacco control with other more strategic ministries. The MoH is relegated to a weaker political position as it is seen as the national budget spender while these other, powerful ministries are the income earners.

*Those who opposed and on the other side are Ministry of industry, trade and agriculture. It is hard. (Respondent 14, Government official)*

### Agendas

In this theme, we present the framings of tobacco and tobacco control which contribute to shaping the tobacco control policy agenda in Indonesia. There are two framings that were derived from our analysis including political economy framing of the tobacco industry and political ideology framing of tobacco control as a foreign agenda.

#### Political economy frame of tobacco: we need the money-we need the employment

Tobacco companies in Indonesia maintain a positive reputation through their perceived contribution to revenue, farming, and employment [24]. Meanwhile, tobacco farming is not a major contributor to the agricultural sector, accounting for only 200,000Ha, less than 1%, of the farming area, [25] yet it is seen as important especially in the tobacco growing provinces. TCEs highlighted that the government is “protecting” the tobacco industry and relies on cigarette revenue, which will significantly undermine tobacco control.

*The government quote unquote wanting to protect the tobacco industry in Indonesia. First, of course the farmers, there is a large tobacco farming sectors in Indonesia, I mean Indonesia is one of the largest tobacco leaves producers in the region and even in the world. So, it is something that, while we don’t want that, Indonesia will be promoting tobacco, it is seen by many politicians as one of those sectors of society, Indonesian society that they feel they need to protect because they are after all the citizens of the country … .The arguments that government has to protect tobacco farmers is not really a valid one, but, that’s how it seen by politician (Respondent 12, International expert)*

#### Sovereignty framing: tobacco control is an “outsider” project

Tobacco control is not yet seen as a priority agenda in Indonesia, even though tobacco use is the fourth leading risk factor of morbidity and mortality, after high blood pressure, dietary risks and high fasting plasma glucose [26]. The TCEs highlighted the consequences of this low attention on tobacco control. For example, the Indonesian commitment to universal health coverage and other sustainable development goals (SDGs) will be significantly stalled by poor tobacco control policy. In 2018, the national health insurance implementing body *(BPJS Kesehatan)* experienced a deficit, partly due to 21.07% spending on catastrophic diseases attributed to tobacco use [27].

*“And it’s especially, is very respectable that Indonesia pushing for universal health care. If you going to push the universal health care you can go bankrupt if you don't get over from the tobacco or at least reduce it drastically. There are some figures from Malaysia a few years ago where they said that 60% of the government budget for health care was due to tobacco use. I mean 60% that's just massive, so if they really have that aspiration to health care for all, I don't see how you gonna do without doing a lot more on tobacco” (Respondent 8, International expert)*

Nevertheless, tobacco control efforts are progressing in Indonesia, both at the national and sub-national level. There is increasing awareness of the need to reduce tobacco use and tobacco control is positively framed in the local media, [6] and more sub-national governments have been gradually adopting smoke-free regulations. However, many politicians still view tobacco control as an “outsider” agenda, partly because foreign donors do support some of the tobacco control advocacy campaigns and research. The tobacco industry supporters argue that foreign interests will negatively impact on Indonesian sovereignty [6]. This argument is grossly unfair when considering that “foreign” tobacco companies control 42.5% of the cigarette market share in Indonesia [28].

*Can you imagine General who is in power now stated that FCTC will ruin Indonesia’s sovereignty, how come? FCTC aim to protect people to be healthy, productive and leap out of poverty, why he/she said destruct sovereignty! (Respondent 2, Academic)*

### Networks

There is a longstanding power imbalance between the tobacco industry network and the tobacco control network in Indonesia, that heavily favours the industry [24]. While some progress in tobacco control advocacy has occurred at both the national and subnational level, it has not shifted the power balance. This third theme comprises sub-themes represent the two networks; namely the established and well-funded tobacco industry networks and the younger and less resourced tobacco control networks.

#### Tobacco industry network: established and well-funded

The tobacco industry is a major industry in Indonesia, with annual cigarette production reaching 342 billion sticks in 2016 [2]. Tobacco company networks are well established and are bound by perceived economic benefits and some are supported by funding from the tobacco industry [24]. These networks include tobacco manufacturers, tobacco leaf growers/farmers, clove farmers, and interested community groups [2]. It is also supported by other players who benefit from tobacco advertising and marketing such as media and advertising companies and sponsorship recipients. The tobacco company networks have stronger links to the policymakers as evidenced by the tobacco interference index score, Indonesia is ranked the highest among South East Asian countries [13].

*Our tobacco control advocates here in Indonesia report that they are regularly learn about meeting in the national level between tobacco executive and policy makers. So, the tobacco companies are very well entrenched. (Respondent 5, International expert)*

*But, don’t be surprise that within the ministries there are lots of TI’s frontline, so, for instance the voice of Ministry of Industry similar to cigarette companies which was stated during the debates (development of PP). (Respondent 4, Advocate)*

TCEs highlighted the fact that tobacco companies infiltrate the policy making process both directly and indirectly. They presented several examples such as significant changes to draft tobacco control regulations, [24] prolonged delays in revising tobacco control laws, and possible interference in a judicial review of broadcasting laws that do not prohibit cigarette ads on television. The government witnesses who spoke positively for tobacco in the judicial review included the television, advertising, farmer, and tobacco industry associations [29].

*The original draft from Parliament commission I, the draft was included prohibition of cigarette commercials, but, when the draft reached baleg (unit within parliament in charge for law making),*^1^*the unit changed it, similar to the current broadcasting law. It only means that someone has a vested interest, only two, the TV association who received the order and the one who gives the order because it is involves a huge amount of money. (Respondent 4 Advocate)*

*Failure of the judicial review on broadcasting law because constitution viewed cigarette as a legal product, so it is legal to being promoted, (but alcohol is also legal?). That is the ambiguous point, why products that are both addictive; both bounded by excise, have different treatment? Is this the tobacco industry interference in the judicative sector? (Respondent 4, Advocate)*

Most ASEAN countries limit industry participation in the policy making process, except Indonesia and The Philippines. These governments allow representatives from industry to take part in committees/advisory groups for public health policy and accept or support legislation drafted in collaboration with tobacco industry [13].

*And of course, the tobacco companies are not helping, they said that they are legal company, they should be stakeholders that need to be consulted. (Respondent 12, International expert)*

#### Tobacco control network: resources and cohesion

In Indonesia, the tobacco control network was pioneered by five diverse groups: well-connected social activists, non-government organizations (NGOs), health professionals, public health and economic researchers and international organisations, including the WHO [24]. The network has evolved and grown to include stakeholders and public health professionals from the sub-national level and now known as the Indonesia Tobacco Control Network (ITCN). Some of the TCEs describe the history of how this group developed, and the challenges it faced at the time, now, and in the future. They highlighted some concerns regarding advocacy progress such as limited resources, contested interests, and the solidity of the network.

*There is internal friction partly for gaining funder sympathy … there is friction between A and B, to claim the credit. There are different views and approaches between groups. … , also, overlapping and uncoordinated programs between organisations. This is weakening the solidity of the group and the tobacco industry knows that. (Respondent 9, Advocate)*

Tobacco control groups have less established connections to policymakers in comparison with tobacco companies [24]. However, the formation of the Mayor Alliance for Tobacco Control, [30] which has contributed to the adoption of smoke free regulations in more than a third of Indonesian districts/cities, is a sign of changing power dynamics. Public support for tobacco control has also been increasing with the involvement of more advocacy groups and government sectors supporting the adoption of smoke free regulations in some Indonesia districts/cities. There is a close and positive connection between the MoH and MWECP, which should widen to include other government departments.

*I think that is the possibility now, especially now in Indonesia in the last five- ten years has developed a very strong civil society movement on tobacco. I think that is real opportunity for them to, to leap forward and not to have to go through the same 40 years process that, that other countries were through to arrive where they are now. (Respondent 8, International expert)*

Internationally, collaborative networks for tobacco control have been growing, this was intensified during the WHO-FCTC negotiations with the establishment of Framework Convention Alliance [31]. The international and regional networks have regularly held meetings and conferences to share policy practices and evidence and conducted trainings and workshops to enhance tobacco control capacity. The TCEs described the support from regional networks such as South East Asia Tobacco Control Alliance (SEATCA) and international partners such as The Union, Vital Strategies, and Campaign for Tobacco Free Kids (CTFK). In 2018, Indonesia hosted the Asia Pacific Conference on Tobacco or Health (APACT) which was considered not only an opportunity to support Indonesian tobacco control advocates, but also to shame the government for its apathy towards tobacco control.

*Well, WHO has been supporting Indonesia to try ratifying the framework convention, I think there was also even at meeting like this (APACT), it sorts of naming and shaming! It’s really embarrassing for Indonesia to be hosting this conference while at the same time its own national government is not actually … hhmm … ratified the framework convention. I can see plenty of countries coming here, countries where it’s really difficult like Mongolia and Laos and Cambodia. (Respondent 1, International expert)*

### Socio-economic factors

In this theme, we described the exclusive positioning of tobacco in Indonesia which contributes to its exception within the law and barriers to policy change. In Indonesia, tobacco is far less regulated when compared to alcohol. The privileged position of tobacco in Indonesia is expressed in the high social acceptability of smoking and the high smoking rate. This special treatment of tobacco is well documented in several Indonesian laws which halt tobacco control progress.

#### Social acceptability of tobacco: cigarettes are legal, and smoking is normal

While politicians position tobacco as a positive product in an economic sense, the community considers tobacco a socially acceptable product. Cigarette smoking has been deeply assimilated into the daily and social life of Indonesians [32]. The term *“uang rokok”* meaning “cigarette money for tipping” is still widely used [24]. Cigarettes have always been part of being a good host during social occasions [32]. These common practices are then manipulated by tobacco companies through aggressive marketing, advertising and promotion, which then preserve and overblow positive views around smoking as part of the culture, as normal and socially acceptable behaviour [32–34].

*Because advertising and promotion are built around messages that talk about independence, being individualistic, being successful, being attractive, being powerful and around also peer acceptance. So, these are messages that are quite preferable by the youths. And so, they feel that smoking is normal, right? (Respondent 12, International expert)*

Moreover, cheap cigarette prices, selling single sticks, and easy access have made cigarettes highly accessible to the population [34, 35]. There is a growing awareness around the negative impact of smoking, but the TCEs stated people generally have vague views on these health issues. Meanwhile, the tobacco industry is seen as a “normal” business and “supportive” through its CSR efforts and community sponsorships [14].

*It is an addictive legal business with long term impact. You smoke then die; it is not like that. The time perspective about risk, we don't have it! That is why insurance is not popular here. Because there is no long-term risk awareness. (People) Can't relate it (cigarette) with cancer, heart disease, because of that time perspective and risk awareness. So, this condition makes cigarette until now is perceived as a “normal” product, treated as normal, which is one source of the failures of all regulations. (Respondent 9, Advocate)*

While tobacco control aims to reduce smoking prevalence, the current high smoking rate itself is a hurdle to adoption of a stronger tobacco control policy [17]. In Indonesia, smoking is considered an acceptable behaviour especially by male, although this is not true of females, as evidenced by the ten-fold gap between male and female smoking rates [4]. There is a global pattern that the willingness to introduce tobacco control measures is higher when smoking prevalence is lower [17].

*Well the barrier the high prevalence among men, it just politically impossible to tell the majority of people, we gonna take away your, take away your cigarette. So, I, I think you gonna have to drive that down in other way (Respondent 8, International expert)*

#### Tobacco is special: pre-emption of other laws

There is evidence of several exceptions given to tobacco compared to alcohol within existing Indonesian laws. Both alcohol and cigarettes are considered addictive substances under the law and as such must be controlled, yet they are treated very differently. Based on broadcasting and press law, alcohol advertisements are banned from mass media platforms, but tobacco advertisements are permitted with few limitations.

*Several laws, 1) Bill No 40/ 1999 on the journalistic/press, 2) Bill No 32, are quite similar. The point is that addictive substance cannot be advertised in print media, TV and electronic media, but, cigarettes can; as long as it does not show the cigarettes. It is not acknowledging that cigarette is addictive. (Respondent 9, Advocate).*

Excise law is further evidence that illustrates how tobacco has a privileged position in comparison with alcohol. Excise tax for alcohol products is set at 80% while for cigarettes, it is capped at a maximum 57% of the retail price, [36] considerably lower than WHO-FCTC standard minimum of 70% [37].

*The excise law gives privilege to tobacco products, the tax differs from other excised goods, its lower-at maximum of 57%, while ethanol and alcohol drink up to 80%. (Respondent 4, Advocate)*

In 2017, cigarettes were taxed at around 50% of the retail price for machine rolled Kretek, 45% for white cigarettes and only 20% for hand rolled Kretek [38]. In September 2019, the Minister of Finance announced a tax increase of 23 and 35% of the retail price from January 2020, without yet specifying the tax increase of each cigarette type [39]. This initiative is positive progress for Indonesian tobacco control especially given the government also plans to simplify the multiple tax tiers, but any further tax increases will be limited by the excise law.

### Ideas and transfer of ideas

The growing and strong evidence of the health consequences of tobacco use drives policy changes; even though there is a notable time lag between the publication of evidence and policy adoption, especially in low-middle income countries [17, 40]. Government adoption of proven tobacco control policy and programme ideas in Indonesia has lagged behind other comparable countries. Indonesia is an outlier in not ratifying the WHO-FCTC when all other Asia Pacific countries are committed.

*I’m trying to be very diplomatic hhm, but it’s really hard to be diplomatic, because the reality is the Indonesia is an outcast, it’s in situation on its own in Asia. … So many things where Indonesia is really lagging behind in term of not fulfilling the pledge to public health and to look after the health of its people. That’s the bottom line, it is not really doing what it should be doing in term of public health. (Respondent 1, International expert)*

The TCE expressed relatively different views on whether the current evidence is enough to support tobacco control advocacy in Indonesia. Most of the experts stated that there is enough evidence around best practice that ideas are ready adaptable for the Indonesian context.

*There is already enough I think, the evidence. It is really up to the presidents and the cabinets to make sure that the policies follow the evidence. (Respondent 12, International expert)*

On the other hand, several experts expressed the need for more local evidence to counter opposition claims that most evidence is from outside Indonesia. There is a need to counter issues that are specific to Indonesia, such as the dominance of Kretek manufacturing and sales.

*… you know that in Indonesia, they will always say, it is overseas (evidence) not in Indonesia. It always come back to denial like that. (Respondent 14, Government official)*

*… we don’t have enough good data to counter (tobacco industry) arguments, so I said to my colleague at Research and Development center (MoH), please help! MoH who have funds should analyse if kretek contain different chemical compare to white cigarette. (Respondent 2, Academic)*

The TCEs also suggested there are signs of obstruction of transfer of ideas, because of the wilful ignorance of policymakers to better understand public health evidence. Some TCEs also observed blocking of the transfer of ideas and evidence to the President. The TCE expressed a positive view of the current President but suggested there are attempts to keep the President unknowledgeable about the real benefits of tobacco control.

*I tried to approach the president from different ways but did not succeed. I heard there was an attempt maybe from the industry, I am not sure, to halt this. The inner circle tries to keep him (the president) not fully understanding. There are structural barriers which make us ineffective in short period. (Respondent 2, Academic)*

## Discussion

Tobacco control policy adoption and implementation is influenced by many complex factors; our study captured factors stalling tobacco control progress in Indonesia from the perspectives of TCEs. Beside the underlying political structure of the decentralised government of Indonesia and its bureaucracy, most factors point to the power of the tobacco industry to influence the tobacco control policy agenda and public perceptions.

### Bureaucracy and decentralized government

Different types of policy structures and power delegations are essential aspects of policy change [17]. The intricate procedures within a government system, lack of coordination, poor accountability and competing interests between government sectors/agencies, contribute to policy adoption and implementation delays [41]. This complex bureaucracy, coupled with power decentralisation, increases vulnerability to corruption [22, 23]. The adoption and implementation of current national tobacco control regulation (PP) and overall tobacco control progress highlights the challenges of these institutional factors.

At a national level, coordination between ministries and government agencies remains a challenge given the opposing political and economic framing of tobacco coupled with the low bargaining position of the MoH. Besides opposition from ministries with direct ties to tobacco industry, overt supporters of the MoH are limited. Ministries that focus on youth and future investment such as Ministry of Youth & Sports, Ministry of Education & Culture, Ministry of Research & Higher Education, have not supported the MoH nor championed tobacco control, choosing instead to accept tobacco industry sponsorship [14]. Recently, the Ministry of Research and Higher Education publicly signed a memorandum of understanding with PT. HM Sampoerna, one of the largest tobacco companies in Indonesia, for research investment and collaboration [42]. Meanwhile, at sub-national level, the PP is not readily implementable and is prone to implementation delay due to the vastness of Indonesia. With substantial autonomy distributed to provincial and city/district level after the adoption of decentralised government system, [20] sub-national governments have not all urgently adopted tobacco control measures, particularly when there is tobacco industry and/ or farming in their jurisdictions.

### Tobacco industry power and influence

Besides actors with decision making powers, such as legislators and government departments, the policy agenda is also contested by actors with non-decision-making powers such as private corporations, in this case the tobacco industry [43]. The tobacco industry is a powerful industry in Indonesia, two cigarette companies are in the top ten largest companies listed in the Indonesian stock exchange [44]. The owners of Indonesian tobacco companies lead the list of the wealthiest Indonesians: Hartono brothers, PT Djarum’s owners and Susilo Wonowidjoyo, the owner of PT Gudang Garam are the richest and second richest Indonesians respectively [45]. Historically, a key tactic used by the tobacco industry to influence the policy agenda is to reframe public health arguments; this helps to shape policy preferences and divert attention from the importance of tobacco control [46, 47]. Tobacco industry money, both directly and indirectly, influences political decision-making and inaction [24, 48]. For example, the cancelation of the annual excise tax increase in Indonesia during the election year 2019, reflects the influence vested interests have on the election cycle and tobacco control.

To date, there is no code of conduct to prevent collaboration between the tobacco industry and government bodies, except the policy adopted internally by The Ministry of Health, nor is there a requirement to report any tobacco industry contribution to political leaders/parties [13]. Under the PP, tobacco company sponsorship is permitted, provided no brand logos are published, however, this has also been circumvented [33]. Advocacy for the adoption of a code of conduct by all government agencies is necessary to minimise this complicit relation. Engaging with the Anti-Corruption Commission (KPK) is an opportunity for tobacco control stakeholders to open communication around reducing tobacco industry interference in policymaking [49].

Despite the stronger structural leverage and political connections to the tobacco company networks, [24] the tobacco control network is evolving. There is increasing public support locally and nationally, intensifying support from regional and international tobacco control networks and from local leaders through Mayor Alliance for Tobacco Control [30]. This positive progress is a sign of changing power dynamics, which should be nurtured by tobacco control advocates. The tobacco control advocates must also enhance their network cohesion and potentially embrace groups within other networks to build a more powerful coalition.

### Tobacco exceptionalism and social norms

While the adoption of the WHO-FCTC as the first international health treaty was celebrated as a significant public health movement, this special attention on the tobacco industry has raised critiques that the exceptionalism given to tobacco is counter-productive to the aim of tackling non communicable diseases (NCDs) more broadly; since the alcohol and junk food industries also promote and sell products which significantly contribute to NCDs [50].

In Indonesia, the near opposite situation exists, where tobacco is far less regulated when compared to alcohol. Besides the strong influence of the tobacco industry on the tobacco control policy agenda in Indonesia, this situation is likely correlated with religious norms in Indonesia, where there is a majority Muslim population. Alcohol consumption is *haram* (prohibited), under Islamic teaching, but views on cigarette smoking remain controversial, with smoking only frowned upon for young children, and women [51]. This controversy is reflected in the different views on smoking of the two major Islamic organisations in Indonesia. While *Muhammadiyah* released a *fatwa* (religious ruling) stating smoking as *haram*, the *Nahdatul Ulama (NU)* declared smoking as *makruh* (discouraged) [51]. The role religious institutions and perspectives play in shaping social norms around tobacco use should also be closely monitored by tobacco control stakeholders.

Additionally, positive norms and social acceptability of smoking is partially driven by omnipresent tobacco advertising, promotion and sponsorship (TAPS), and the high availability and accessibility of cigarettes among Indonesians. The current PP does not adequately restrict tobacco marketing and reduce TAPS exposure, especially among young people as reflected by the increasing smoking rate among young people [4]. Revision of the PP to optimally adopt TAPS ban outlined by in the WHO-FCTC article 13 is urgently required [52].

### Limitations

Our study is subject to some limitations. The study only considers TCEs perspectives on factors that are stalling tobacco control in Indonesia and as such only covers the proponent side of tobacco control and may not reflects the views of other sectors. A future study could explore stakeholder perspectives from both proponents and opponents of tobacco control.

## Conclusion

Big tobacco has substantially influenced both the policy decision process and public perceptions of its importance to the economy. WHO-FCTC ratification reluctance and the adoption of only partial tobacco control regulation is clear evidence of the power imbalance between the tobacco industry and tobacco control. The adoption of the PP was a significant stepping stone for tobacco control in Indonesia, but its overdue development and delayed implementation signifies the ongoing struggle for tobacco control stakeholders to navigate complex and corrupt bureaucracies and powerful commercial interests. Engaging with the Anti-Corruption Commission (KPK) is an opportunity for tobacco control stakeholders to open communication around reducing tobacco industry interference in policymaking [49]. Advocacy for the adoption of a code of conduct and revision of the PP to address loopholes regarding stakeholder responsibility should be a priority.

The Indonesian government could readily minimise the structural leverage of tobacco companies by adopting the WHO-FCTC and immediately fully implementing both Article 5.3 on tobacco industry interference, [53] and Article 13 on tobacco advertising bans [52]. The tobacco control advocates must also enhance their network cohesion and potentially embrace groups within other networks to build a more powerful coalition. Engagement with policymakers could be improved by framing tobacco use as economic issue as well as a health concern. The role religious institutions and perspectives play in shaping social norms around tobacco use should also be closely monitored by tobacco control stakeholders.

## Supplementary information

**Additional file 1.** Supplementary file 1. Interview Guideline.

## Data Availability

The data are not publicly available due to containing information that could compromise research participant privacy/consent, but the data that support the findings of this study are available on reasonable request. Please contact the corresponding author.

## Abbreviations

ASEAN: Association of South East Asia Nation
APACT: Asia Pacific Conference on Tobacco or Health
*BPOM*: *Badan Pengawas Obat dan Makanan* (Food and Drug Monitoring Agency)
*BPJS-Kesehatan*: *Badan Penyelengara Jaminan Sosial-Kesehatan* (Health Insurance Implementing Body)
CSR: Corporate Social Responsibility
CTFK: Campaign for Tobacco Free Kids
FCTC: Framework Convention on Tobacco Control
ITCN: Indonesia Tobacco Control Network
*KPI*: *Komisi Penyiaran Indonesia* (Indonesia Broadcasting Commission)
*KPK*: *Komisi Pemberantasan Korupsi* (Anti-Corruption Commission)
MoH: Ministry of Health
MWECP: Ministry of Women Empowerment and Child Protection
NCDs: Non-Communicable Diseases
NGOs: Non-Government Organizations
*PP*: *Peraturan Pemerintah* (Government Regulation)
SEATCA: South East Asia Tobacco Control Alliance
SDGs: Sustainable Development Goals
TAPS: Tobacco Advertising, Promotion and Sponsorship
TCEs: Tobacco Control Experts
WHO: World Health Organization

## Footnotes

1 http://www.dpr.go.id/akd/index/id/Tentang-Badan-Legislasi
