# Supplementary material for: Why is tobacco control progress in Indonesia stalled? - a qualitative analysis of interviews with tobacco control experts
Source: BMC Public Health. 2020 Apr 19;20:527. doi: 10.1186/s12889-020-08640-6 (PMC7168953; doi:10.1186/s12889-020-08640-6)
Supplement: Supplementary file 1 — Additional file 1. Supplementary file 1. Interview Guideline. [file 12889_2020_8640_MOESM1_ESM.docx]

TOBACCO CONTROL EXPERTS’ OPINION ON

FACTORS STALLING TOBACCO CONTROL PROGRESS AND

THE FUTURE OF TOBACCO ADVERTISING

AND MARKETING REGULATION IN INDONESIA

**Interview guideline**

Greetings and explanation on the purpose of the interview and how the interview will be conducted. Clarifying that the respondent has read and understand the participant information statement (PIS) that was sent via email/instant message. Providing time to read the participant consent form (PCF) and providing consent.

**Question guide**

1. What is your opinion on current smoking rate in Indonesia, including among young people?
2. What factors are affecting this smoking rate?
3. How much do you think tobacco marketing and tobacco advertisements, promotion and sponsorship (TAPS) influence the smoking rate and smoking among young people?
4. Ideally, what measures should be taken to control tobacco marketing and tobacco advertisements, promotion and sponsorship (TAPS)?
5. Who should be responsible for taking those measures?
6. Why hasn’t Indonesia adopted these ideal measures? What are the barriers?
7. In your opinion, what should be done to overcome these barriers?
8. Is there any factor that would enable or support the achievement of the ideal measures?
9. Please suggest, what strategies should be taken by the government in national and sub-national level to achieve these ideal measures?
10. Besides regulating the TAPS, what do you think about regulation tobacco retailing?
11. What are enablers and barriers for regulating tobacco retailing?
12. What other strategies other than mentioned above will support this retailing policy?
13. When do you think these measures could be achieved?
14. Anything you wish to add?
